# Weathering Stability and Durability of Birch Plywood Modified with Different Molecular Weight Phenol-Formaldehyde Oligomers

**DOI:** 10.3390/polym13020175

**Published:** 2021-01-06

**Authors:** Juris Grinins, Vladimirs Biziks, Brendan Nicholas Marais, Janis Rizikovs, Holger Militz

**Affiliations:** 1 Latvian State Institute of Wood Chemistry, 27 Dzerbenes Str., LV-1006 Riga, Latvia; janis.rizikovs@gmail.com; 2 Georg-August University of Goettingen, Wood Biology and Wood Products, Büsgenweg 4, 437077 Göttingen, Germany; vbiziks@gwdg.de (V.B.); bmarais@uni-goettingen.de (B.N.M.); hmilitz@gwdg.de (H.M.)

**Keywords:** birch plywood, molecular weight, phenol-formaldehyde resin, soft-rot, weathering stability

## Abstract

This study investigated the effect of phenol-formaldehyde (PF) resin treatment on the weathering stability and biological durability of birch plywood. Silver birch (*Betula pendula*) veneers were vacuum-pressure impregnated with four different PF resins with average molecular weights (M_w_) of 292 (resin A), 528 (resin B), 703 (resin C), and 884 g/mol (resin D). The aging properties of PF resin modified birch plywood were analyzed using artificial weathering with ultraviolet (UV) light, UV and water spray, and weathering under outdoor conditions. The same combinations of PF-treated plywood specimens were then tested in soil-bed tests to determine their resistance against soft-rot wood decay. It was not possible to compare weathering processes under artificial conditions to processes under outdoor conditions. However, the weathering stability of birch plywood treated with PF resins A, B, and C, scored better than plywood treated with commercial resin D (regardless of solid content concentration [%]). Results from unsterile soil bed tests showed improvements in resistance to soft-rot wood decay compared to untreated plywood and solid wood. Mass loss [%] was lowest for birch plywood specimens treated with resin of highest solid content concentration (resin D, 20%). Provisional durability ratings delivered durability class (DC) ratings of 2–3, considerably improved over untreated solid wood and untreated birch plywood (DC 5).

## 1. Introduction

Wood is increasingly being used for outdoor applications, yet it is still limited by complex wood-water-ultraviolet (UV) light interactions. During weathering, wood is cycling through wet and dry states, thus inducing repeated swelling and shrinkage and generating tension stresses. Wood responds to these wetting-drying stresses through creeping and surface cracking. Once the stresses exceed the fracture strength of wood, it has a tendency to develop longer and deeper cracks at later stages [1]. These cyclic changes in moisture content and dimensions are most pronounced at the wood surface, which is directly exposed to rain, humidity and sunlight (UV and visible light). Moreover, UV light exposure intensifies crack formation because photodegradation weakens the wood surface and degrades its microstructure [2]. The presence of cracks and other defects is thus a major drawback and may reduce its service life, market value and mechanical strength. These defects also lead to increased water uptake, thus producing optimal moisture conditions for wood-decay fungi to attack [3].

Silver birch (*Betula pendula*) plywood is widely used in construction, interior and exterior decoration, vehicle construction, sports equipment, furniture, packaging materials, and toy production. However, its application in outdoor, high humidity conditions is limited due to poor biological durability (durability class 5 according to EN 350-1:2016 [4]). The effect of wood degrading fungi, humidity and UV light, essentially impair the technical properties of plywood through weakening of the bonded veneers, which in-turn weaken mechanical strength and deteriorate the surface finish. Moisture easily penetrates into veneer layers, which causes swelling and characteristic waves on the plywood surface. Water uptake potential can be decreased by covering the plywood with a hydrophobic (water repelling) laminate. Melamine and phenolic resins are mostly used for the production of resin laminates, which are subsequently hot-pressed onto one or both sides of the finished plywood board surfaces. Coating plywood protects the top veneer layer from direct contact with water and UV light, but when the upper laminate coating is damaged, the inner veneer layers can swell, provoking noticeable surface failure and promoting attack by biodegradation agents. Most wood species used for plywood production have poor resistance to swelling, biodegradation and UV weathering under outdoor and high humidity conditions. Therefore, when using plywood in conditions with high humidity, where regular wetting is possible, it is necessary not only to cover it from the outside with a hydrophobic laminate, but also to treat the single veneers constituting the entire plywood board. Prolonging the service life of wood and wood-based products results in a positive effect on greenhouse gas emissions by storing biomass carbon for longer periods [5].

Wood modification alters the material properties to a greater extent than preservative treatment, and the magnitude of changes depends on the applied modification method [6]. Wood modification can simultaneously overcome several weaknesses of wood, such as poor dimensional stability, low decay resistance, high equilibrium moisture content, and aesthetical issues such as optical appearance can be diversified and enhanced. Wood impregnated with most thermosetting resins causes changes in colour [7]. Compared to untreated wood, acetylated wood [8,9], glutaraldehyde treated wood [10], DMDHEU- and melamine- treated wood [7] exposed to accelerated or outdoor weathering develops fewer cracks because of its improved dimensional stability. Surface discoloration (graying) and crack development during longer exposure times is reduced, whereas in the case of thermal treatment, the rate of graying and crack development is the same or even faster than that of untreated wood [3,11]. In contrast, the phenol formaldehyde (PF) treated boards remained darker, ranging from light brown to dark brown. PF resin turns wood red-brown, due to differences in pH, because wood is acidic and resole PF resins are alkaline [12]. This change in optical appearance depends on both the resin type and the average molecular size of the PF resin oligomers used for treatment. PF resin acts as a UV absorber for the photostabilization of wood [13]. PF resin is also an antioxidant, which may also impact its ability to photostabilize wood [14]. Kielmann and Mai [15,16] have concluded that the surface of PF-treated wood without a coating has improved resistance against photodegradation compared to the surface of N-methylol melamine (NMM)-treated wood because PF inhibits lignin degradation. The resistance of wood treated with low molecular weight PF resin to weathering can be improved by increasing the concentration of PF resin and by combining it with a water soluble hindered amine light stabilizer [17]. PF resins could be modified with ferric chloride and a mixture of ferric sulphate and hydrolysable polyphenols to darken the colour of European beech wood (*Fagus sylvatica* L.) and enhance colour stability [18]. Although a proper improvement in dimensional stability and biological durability and a considerable reduction in water sorption are attained, the appearance of the treated wood still undergoes considerable changes during weathering. Therefore, over the past decade, the combined approach of coating chemically treated wood has become an increasing point of interest as a way of increasing wood service life.

As mentioned, outdoor wooden components are subjected to a variety of biotic and abiotic degradation factors. Additionally, wood used in soil contact is of particular risk due to the permanent to semi-permanent presence of moisture [19]. Important considerations for the successful proliferation of wood-decaying fungi include a carbon substrate, moisture, temperature, and oxygen [20]. Various wood decaying fungi; brown-, white-, and soft-rot fungi, can all be found on wood utilized in-ground, but these decay types can vary significantly, not only in frequency and spatial distribution, but also in combinations from one site to the next [21,22], and with decay progress [23]. Soft-rot seems to be able to cope with high soil moisture content (MC_soil_) better than brown- and white-rot fungi, and continues to remain active over a broader temperature range (T_soil_) compared to brown- and white-rot fungi [21,24,25].

In the pursuit of new techniques for improved wood protection, a rapid assessment of the technique’s effectiveness can be attained through laboratory tests. Such tests deliver results quickly and thus form the basis for the decision for further test steps. It is of the greatest interest that the predictions obtained from these tests can be transferred to practice (field tests) with a high degree of certainty. One possibility to better assess the risk factors that determine wood degradation when used in contact with soil is to test the wood in-field and in contact with soil. This test in an option when assessing the behavior of wood preservatives by DIN EN 252:2015 [26]. However, depending on the duration and characteristics of the vegetation periods, it may take several years before results from this type of test are available. Since this method requires a lot of time, the use of pure-culture basidiomycete tests such as CEN/TS 15083-1:2005 [27] and unsterile soil bed tests such as CEN/TS 15083-2:2005 [28] under controlled laboratory conditions are often employed. The results from these tests are designed to complement each other in combination with longer-term field tests using specimens of larger dimension. Newly developed wood preservative and modification techniques can therefore be tested to deliver preliminary durability ratings.

This study compared the color changes of a developed birch plywood modified with different PF resins after weathering under artificial conditions with UV light only, and UV light and water spraying, and under real outdoor conditions. Additionally, so called terrestrial microcosms (TMC) consisting of unsterile organic soil were used to test the resistance of the developed birch plywood against wood decay by soft-rot fungi. This study impregnated birch wood veneers with PF resin solutions of different solid content concentrations and PF oligomer sizes, to understand the effect on dimensional stability, weathering performance, and biological durability. Theoretically, the photostability and resistance to wood decaying fungi of the wood material treated with low molecular weight PF resins should be improved. Also, increasing the concentration of PF resin should improve the weathering and biological durability of the treated wooden material. Criteria for birch veneer treatment was based on minimum WPG requirements to achieve the maximum improvement in properties, therefore to limit PF resin load in the wood material.

## 2. Materials and Methods

### 2.1. Weathering Stability Test

#### 2.1.1. PF Resin Synthesis

For the synthesis of resin A, B, and C, phenol was hydroxymethylated under alkaline reaction conditions, whereby the molar ratio of formaldehyde/phenol/sodium hydroxide was 2.0/1.0/0.2. During the synthesis of each resin, a measured amount of phenol and aqueous sodium hydroxide solution was weighed out in a 4-neck laboratory reactor (1 L) equipped with a thermometer, dropping funnel, reflux condenser and stirrer. Some ethanol was also added in order to maintain a homogeneous reaction. The 4-neck reactor was submerged in a thermostatic water-bath. As soon as the temperature in the flask reached the necessary synthesis temperature, the aqueous formaldehyde solution was added slowly via a drip over a 25–30 min period. The reaction temperature (65, 75, 85 °C) was kept constant during the entire reaction period (2 and 4 h). The resol synthesis was ended by cooling the reactor with cold running water and allowing the resol to cool down to 20 ± 3 °C. Resin D was provided by Prefere Resins Germany GmbH (Erkner, Germany). The different characteristic parameters of the synthesized PF resins are listed in Table 1.

#### 2.1.2. Resin Characterisation

The dynamic viscosity of liquid PF resins was determined by a Fungilab Viscolead Adv meter (Fungilab S.A., Barcelona, Spain) equipped with a suitable spindle. The non-volatiles content (solid content) was determined according to DIN EN ISO 3251:2019 [29]. The pH value was determined using a digital pH meter (GPH 114 Greisinger, Regenstauf, Germany) by inserting the pH meter electrode into the PF resins. The pH meter was calibrated with buffer solutions at pH 4.0 and 10.0 prior pH measurements. Free formaldehyde content was determined by the hydroxylamine hydrochloride method according to DIN EN ISO 9397:1997 [30].

For gel permeation chromatography (GPC) analysis, a 1260 Infinity system (degasser, isocratic pump, automatic liquid sampler, heatable column compartment, RID, MWD @ 280 nm, Agilent (Santa Clara, CA, USA) was used, where: column: 3 × PLgel 5 µ (50 Å, 100 Å, 1000 Å), 7.5 × 300 mm; solvent: tetrahydrofuran (THF); flow rate: 0.6 mL/min; flow rate marker: acetone; calibration: polystyrene standard.

Approximately 40 mg of resin was dissolved in 5 mL of THF. If the resin did not completely dissolve, it was sonicated with slow addition of H_2_SO_4_ (5% in methanol) until neutral. If the resin was dissolved, but precipitate from additives (such as salts) remained, the mixture was filtered with a syringe filter.

#### 2.1.3. Treatment of Veneer Material

Air-dried veneer sheets of silver birch wood (300 × 300 × 1.5 mm^3^ and 400 × 400 × 1.5 mm^3^ L × R × T) were prepared for impregnation. Oven-dry mass was determined after drying at 103 ± 2 °C for 24 h. Previous experiments suggested that treatment of birch wood with commercial PF resin solutions of 10% concentration delivered weight percentage gain (WPG) of 9–12%, which subsequently improved dimensional stability and allowed for a provisional durability class (DC) rating of 1 against decay fungi [31,32]. Veneers (300 × 300 × 1.5 mm^3^) were conditioned to 5–6% moisture content and impregnated with 10% solid content concentration solutions of PF resins A, B, and C. Impregnation was carried in a 340-litre chamber produced by Wood Treatment Technology (WTT, Grindsted, Denmark). Veneers (n = 40) were placed in a tub filled with the resin solution. The veneers were prevented from floating using a mesh grid and heavy weight. Impregnation was carried out in two steps. The first, vacuum step (1 h, 0.1 bar of pressure), was used to ensure the free air was purged from the specimens. The chamber was then pressurized to ensure sufficient diffusion of the PF oligomers into the wood cell walls (1 h, 4 bars of pressure). The veneers were then removed from the impregnation chamber and measured for solution uptake. The remaining resin solution was drained from the tub and the veneers were positioned vertically to allow excess resin solution to drip off. After impregnation, veneers were oven dried to 4–6% moisture content, with moderate air circulation and air exchange for 72 h using incrementally rising temperature intervals from 30–50 °C. A subset of PF resin impregnated veneers from each resin impregnation treatment was measured for WPG. These veneers were cured at 140 °C for 1 h. The WPG was calculated to assess the amount of PF resin in the veneers. The average WPG was calculated for each treatment according to Equation (1) below:

(1) $$\mathrm{WPG}=\frac{\left(M_{1}-M_{2}\right)}{M_{2}}\times 100$$

where:

- WPG is the weight percentage gain [%];
- $M_{1}$ is the oven-dried mass of the modified wood specimens [g];
- $M_{2}$ is the oven dry mass of the unmodified wood specimens [g].

Commercial resin D was used to evaluate the behaviour of different loading of PF resin in veneers. Therefore, before being used for impregnation, the stock solution of the PF resins D was diluted with distilled water to 10, 15 and 20% solid content concentration. Veneers (400 × 400 × 1.5 mm^3^) were conditioned to 5–6% moisture content and impregnated with PF resin solutions in a 1000-litre impregnation chamber at the University of Göttingen. The impregnation and drying parameters were set the same as for veneers of 300 × 300 × 1.5 mm^3^. The WPG was calculated to assess the amount of PF resin in the veneers. Veneer WPG after impregnation and curing for both veneer dimensions is shown in Table 2 below.

#### 2.1.4. Plywood Production

Standard PF adhesive (sourced from plywood industry partners) was applied to the veneer sheets in preparation for pressing into plywood (nine layers). PF adhesive viscosity was 380 mPas, solid content 44.5%, free formaldehyde content <1% and pH 12.6. A quantity of 150 g/m^2^ was applied to one surface of eight of the nine plywood sheets constituting a 9-layer plywood board. After adhesive application, veneers were pre-dried at room temperature for 15 min (adhesive open time) before assembling nine individual veneer sheets in a crosswise, perpendicular fashion in preparation for pressing. Assembled sheets were then pressed in a hot press (Joos LAP 40, Gottfried Joos Maschinenfabrik GmbH & Co. KG, Pfalzgrafenweiler, Germany) at 140 °C and 1.5 N/mm^2^ for 20 min (90 s/mm) to deliver a plywood board with thickness of approximately 11 mm. Thereafter specimens were prepared for artificial weathering, outdoor weathering and unsterile soil-bed tests with dimensions of 150 × 70 × 11 mm^3^, 110 × 40 × 11 mm^3^ and 100 × 10 × 11 mm^3^, respectively.

#### 2.1.5. Artificial Weathering Tests

Artificial weathering tests were performed in a QUV accelerated weathering tester, (Q-Lab Europe, Ltd., Farnworth Bolton, England) equipped with UVA-340 type fluorescent lamps. Three plywood specimens (150 × 70 × 11 mm^3^) were used for each resin treatment. The lamps provided a good simulation of sunlight in the short wavelength region; from 295 nm to 365 nm, with a peak emission at 340 nm. The UV radiation flux density at 340 nm was 0.89 W/m^2^ and the chamber temperature throughout the test was kept constant at 60 °C. The intensity of the full UV spectrum’s (290–400 nm) irradiation was 21.5 W/m^2^. In the study, two different artificial weathering tests were carried out. The first test involved only UV irradiation. This test was regularly suspended to measure the change in colour of the specimens. The total duration of the test was 360 h. The second artificial weathering test involved both UV irradiation and water spray. The test involved the following steps; 2.5 h of UV radiation at the same conditions as described earlier, followed by 30 min of water spray. In total, 60 cycles were preformed to reach an exposure time of 180 h from which 150 h accounted for UV irradiation. Colour measurements after both weathering methods was performed.

#### 2.1.6. Surface Colour Measurements

Colour of the specimens was measured with a CM-2500d spectrophotometer (Konica Minolta, Ramsey, NJ 07446, USA) and expressed according to the CIELAB three-dimensional colour system. On each of the specimen, five locations were randomly chosen and marked. For the marked locations, the colour was measured before and after the weathering tests as well as during the test after 2, 4, 8, 16, 24, 48, 120, 192, 264 and 360 h. The colour was measured to evaluate the discolouration caused by weathering. The total colour change ΔEab was calculated according to the Equation (2) below. L* is a lightness parameter, a* is a chromaticity parameter which represents red-green coordinates and b* is a chromaticity parameter which represents yellow-blue coordinates:

(2) $$∆\mathrm{Eab}=\sqrt{{\left(L_{x}^{*}-L_{o}^{*}\right)}^{2}+{\left(a_{x}^{*}-a_{o}^{*}\right)}^{2}+{\left(b_{x}^{*}-b_{o}^{*}\right)}^{2}}$$

where:

- $L_{o}^{*}$, $a_{o}^{*}$, $b_{o}^{*}$ is the value on coordinate axis for the specific parameter at the beginning;
- $L_{x}^{*}$, $a_{x}^{*}$, $b_{x}^{*}$ is value on coordinate axis for the specific parameter after weathering.

#### 2.1.7. Outdoor Weathering and Fungal Tests

Six replicates of all PF resin treated plywood with dimensions of 110 × 40 × 11 mm^3^, along with untreated specimens, were used. For half of the specimens (3 from each impregnation treatment), the side edges were coated with urethane alkyd paint (brushed on application, three coats). According to EN 152:2011 [33], the samples were placed in an aluminium rack at 45°, one meter above the ground, facing the south direction with most severe weather conditions. Microorganisms were allowed to attack the specimens. The test site in the courtyard of Latvian State Institute of Wood Chemistry (Riga, Latvia) was free of vegetation, shade and extreme environmental conditions. The test lasted for 3 months, from 19 June to 21 September 2020 and weather data are listed in Table 3. 

Mould and blue stain growth was evaluated once per month by stereomicroscopy (M8, Leica, Wetzlar, Germany) and digital photography (2 MB) according to the rating scale 0–4: 0—clean, 0% attack; 1—trace, ≤5% growth; 2—slight, 6–25% growth; 3—medium, 26–50% growth; 4—severe, >50% growth. Colour change measurements were also performed once per month.

### 2.2. Unsterile Soil-Bed Test: Resistance against Soft-Rot Wood Decay

Terrestrial microcosms (TMCs) in accordance with CEN/TS 15083-2:2005 were utilised to test the resistance of the developed plywood material against soft-rot wood decay. The standard stipulates that a natural topsoil or a fertile loam-based horticultural soil substrate is used, with pH 6–8, and no additives. The soil should have a WHC_soil_ of 20–60%, MC_soil_ equal to 95%WHC_soil_, and the test should be conducted in a dark, climate-controlled room set to a temperature of 27 °C and relative humidity of 65%.

#### 2.2.1. Soil Substrate

The soil substrate was a horticultural compost produced at the forest botanical garden at the University of Göttingen’s North Campus. The compost comprised of fallen leaves and cuttings from grass and trees. Soil was passed through a sieve with nominal aperture size of 8.5 mm. WHC_soil_ was then determined according to the ‘cylinder sand bath method’ according to ISO 11268-2:2012 [34]. The test deviated from the standard in that no silica sand was added to the soil in order to reduce the soil’s water-holding capacity. Silica sand acts to standardize and reduce the soil’s inoculum potential to attain reproduceable wood decay results which serves as a provisional durability rating.

#### 2.2.2. Determination of the Soil-Water Holding Capacity (WHC_soil_)

Soil was inserted into polyethylene cylinders 10 cm long with 4 cm diameter. The bottoms of the cylinders were covered with a fine polymer grid and filter paper (MN 640 W 70 mm). All cylinders were filled with soil to a height of 5–7 cm and saturated in an 8 cm high water bath for 3 h. After the saturation period, the cylinders were placed on a water saturated sand bath for 2 h to allow unbound water within the soil-filled cylinders to drain to reach the equivalent of field capacity. The soil samples were then weighed wet, as well as after oven-drying at 103 ± 2 °C for 24 h. WHC_soil_ [%] was calculated according to Equation (3) below. The compost soil batch deliver WHC_soil_ of 105%:

(3) $${\mathrm{WHC}}_{\mathrm{soil}}=\;\left(\frac{m_{s}-m_{0}}{m_{0}}\right)\times 100$$

where:

- ${\mathrm{WHC}}_{\mathrm{soil}}$ is the soil water-holding capacity [%];
- $m_{s}$ is the saturated soil mass [g];
- $m_{0}$ is the oven-dry soil mass [g].

#### 2.2.3. Determination of the Soil Moisture Content (MC_soil_)

In order to ensure an equal quantity of soil was used across all TMC boxes, measurements of soil moisture content (MC_soil_) were used. The soil quantity decided on for all TMC boxes was based on the based on the oven-dry mass of the soil [g], and was dependent on the box’s dimension. Each TMC box was filled to ensure a soil height of approximately 12 cm, to this end the oven-dry soil mass decided on for all TMC boxes amounted to 6000 g. The plastic TMC boxes were initially weighed in dry, empty state. Then, TMCs were over-filled with soil (i.e., >12 cm in height), with the box (including soil) weighed again to calculate the mass [g] of the soil component added to the box. Five MC_soil_ samples of 50 g each were then taken from multiple locations throughout the TMC box to ensure an average, but homogenously distributed MC_soil_ measurement was attained. Soil samples were weighed to the nearest 0.001 g, oven-dried at 103 °C for 24 h, and weighed again. MC_soil_ was calculated according to Equation (4) below. Once a representative MC_soil_ measurement was attained, a rearrangement of Equation (4) below was carried out to calculate the quantity of ‘wet soil’ ($m_{w}$) required to be removed from the TMC box to attain 6000 g of oven-dry soil ($m_{0}$):

(4) $${\mathrm{MC}}_{\mathrm{soil}}=\left(\frac{m_{w}-\;m_{0}}{m_{0}}\right)\times 100$$

where:

- ${\mathrm{MC}}_{\mathrm{soil}}$ is the soil moisture content [%];
- $m_{w}$ is the wet soil mass [g];
- $m_{0}$ is the oven-dry soil mass [g].

#### 2.2.4. Preparation of Soil Substrates to Reach Target Soil Moisture Content (MC_soil,target_) 

Once 6000 g of oven-dry soil was filled into each of the six TMC boxes, MC_soil_ for each TMC was set to equal 95% of the measured WHC_soil_, expressed as 95%WHC_soil_. In order to understand the quantity of distilled water required to reach the MC_soil_ equal to 95%WHC_soil_, a target soil moisture metric MC_soil,target_ [%] was defined. Equation (5) below was used to calculate the mass [g] in distilled water required to add to the soil mixture to reach MC_soil,target_. Distilled water was subsequently added to each of the TMCs to reach MC_soil,target_. To account for losses in MC_soil_ resulting from fungal activity and evaporation, rewetting to MC_soil,target_ occurred once per week throughout the 24-week incubation period:

(5) $$m_{\mathrm{water}}=\left(\frac{{\mathrm{MC}}_{\mathrm{soil},\mathrm{target}}-{\mathrm{MC}}_{\mathrm{soil},\mathrm{current}}}{100}\right)\times \;m_{\mathrm{total},\mathrm{dry}}$$

where:

- $m_{\mathrm{water}}$ is the mass of distilled water to add to the soil mixture [g];
- ${\mathrm{MC}}_{\mathrm{soil},\mathrm{target}}$ is the target soil moisture content [%];
- ${\mathrm{MC}}_{\mathrm{soil},\mathrm{current}}$ is the current moisture content of the soil mixture before adding any additional water [%];
- $m_{\mathrm{total},\mathrm{dry}}$ is the oven-dry mass of the total soil mixture [g].

#### 2.2.5. Preparation and Exposure of Wood Specimens 

As already mentioned, some aspects of this TMC test against soft-rot wood decay deviated from the standard CEN/TS 15083-2:2005 [28]. One deviation was the inoculum aggressiveness of the soil material used, the other deviation, which also influenced the decision to use a more aggressive soil was the specimen dimension. Plywood boards of birch with nine veneer layers were prepared. The final height (thickness) of the 9-layer plywood boards amounted to 11.5 mm. Thereafter, individual specimens were prepared from the larger plywood boards to final dimensions of 10 × 11.5 × 100 mm^3^. 

Before specimens were prepared from the larger plywood boards, the boards were conditioned to wood moisture content (MC_wood_) of 12 ± 2%. MC_wood_ was tested using a 2-pronged electrical resistance moisture content measuring device. Specimens were then prepared from strips of the boards, with a cross-section of 10 ± 0.1 mm × 11.5 ± 0.1 mm (board thickness). Transverse cuts of the cross-section delivered sharp edges and a fine-sawn finish to the end-grain surface, with a final specimen length of 100 ± 1 mm. All specimens were free from obvious defects such as cracks, decay and discolouration. 

After specimen preparation, all specimens were oven-dried at 103 °C for 24 h and weighed for oven-dry mass to the nearest 0.001 g. Prior to soil exposure, all specimens were again conditioned to MC_wood_ of 12 ± 2% (confirmed by Equation (6) and buried 4/5 of their length into the soil substrate with 38 specimens per TMC box. For control (plywood, birch and beech solid wood) 30 replicate specimens were used with three specimen removal intervals (16, 20, 24 weeks). For each PF-treated plywood type 20 replicate specimens were used, with two specimen removal intervals (16 and 24 weeks). After soil exposure, specimens were removed, cleaned of remaining soil and again oven-dried at 103 °C for 24 h. Specimens were then weighed again to the nearest 0.001 g with oven-dry wood mass loss (ML_wood_) calculated according to Equation (7) below. Mean ML_wood_ and standard deviation of mean ML_wood_ was calculated according to Equation (8) and Equation (9) below:

(6) $${\mathrm{MC}}_{\mathrm{wood}}=\left(\frac{m_{3}-\;m_{2}}{m_{2}}\right)\times 100$$

where:

- ${\mathrm{MC}}_{\mathrm{wood}}$ is the wood moisture content, [%];
- $m_{3}$ is the wood specimen’s mass after TMC exposure, [g];
- $m_{2}$ is the wood specimen’s oven-dry mass after TMC exposure, [g].

Oven-dry mass loss (ML_wood_) of wood was calculated according to Equation (7) below:

(7) $${\mathrm{ML}}_{\mathrm{wood}}=\left(\frac{m_{1}-\;m_{2}}{m_{1}}\right)\times 100$$

where:

- ${\mathrm{ML}}_{\mathrm{wood}}$ is the wood specimen’s oven-dry mass loss [%];
- $m_{1}$ is the wood specimen’s oven-dry mass before TMC exposure [g];
- $m_{2}$ is the wood specimen’s oven-dry mass after TMC exposure [g].

Mean ML_wood_ was calculated according to Equation (8) below:

(8) $${\mathrm{mean}\;\mathrm{ML}}_{\mathrm{wood}}=\frac{1}{n}\overset{n}{\underset{i=1}{\sum }}x_{i}$$

where:

- ${\mathrm{mean}\;\mathrm{ML}}_{\mathrm{wood}}$ is the arithmetic mean of the oven-dry mass loss of the sample population;
- $x_{i}$ is the oven-dry mass loss (ML_wood_) of each individual wood specimen in the sample population;
- n is the total number of wood specimens in the sample population.

Standard deviation of mean ML_wood_ was calculated according to Equation (9) below.

(9) $$s=\sqrt{\frac{\sum_{i=1}^{n}\left(x_{i}-\;\overset{-}{x}\right)}{n-1}}$$

where:

- s is the standard deviation of the sample population;
- $x_{i}$ is the oven-dry mass loss (ML_wood_) of each individual wood specimen in the sample population;
- $\overset{-}{x}$ the mean oven-dry wood mass loss (mean ML_wood_) of the sample population [g];
- n is the total number of wood specimens in the sample population.

#### 2.2.6. Calculation of x-Value towards Provisional Durability Rating

According to CEN/TS 15083-2:2005 [28], the percentage oven-dry mass loss of the tested wood specimens is used to determine the resistance of hardwood test timbers to wood decay by soft-rotting fungi. The calculation of the x value based on the median oven-dry mass loss of the treated test specimens and the untreated reference specimens is used to define a provisional durability class (DC) rating. However, DC ratings do not equal use class ratings (like those covered in EN 335:2013 [35]. Use classes, and the combination of use classes and DC to assess wood suitability for a particular application are addressed in EN 460:1994 [36] and prEN 460:2019 [37], respectively. Equation (10) below was used to calculate the x value:

(10) $$x=\;\frac{{\mathrm{median}\;\mathrm{ML}}_{\mathrm{wood}}\;\mathrm{of}\;\mathrm{the}\;\mathrm{various}\;\mathrm{treated}\;\mathrm{test}\;\mathrm{specimen}\;\mathrm{group}}{{\mathrm{median}\;\mathrm{ML}}_{\mathrm{wood}}\;\mathrm{of}\;\mathrm{reference}\;\mathrm{test}\;\mathrm{specimens}}$$

where:

- x is the x value used for interpretation in a provisional durability rating scale;
- ${\mathrm{ML}}_{\mathrm{wood}}$ is the oven-dry mass loss of the relevant wood specimen.

## 3. Results and Discussion

### 3.1. Artificial Weathering (UV Only)

**Figure 1:**
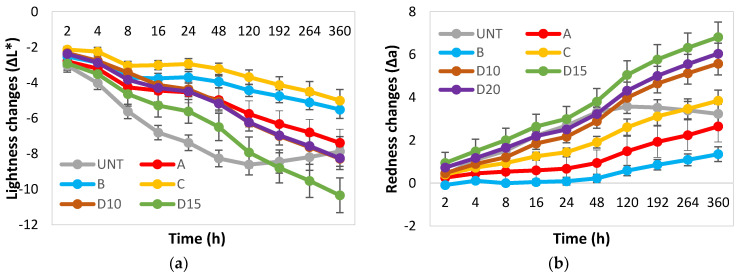

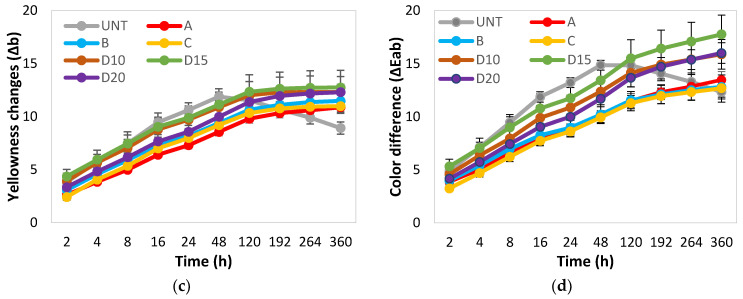
Changes in colour of untreated (UNT) and PF resin treated plywood after exposure to UV light during 360 h in QUV camera: (**a**) Changes in the CIE parameter ΔL* (lightness); (**b**) Changes in the CIE parameter Δa (red-green); (**c**) Changes in the CIE parameter Δb (yellow-blue); (**d**) colour difference parameter ΔEab.

There is a demand among end users for new plywood products with predictable, long-term aesthetic properties. Plywood products with improved colour stability naturally have an advantage over competitors. In order to evaluate how UV radiation affects the PF resin treated plywood developed in this study, colour parameters were evaluated at different exposure intervals. The results presented in Figure 1 show the colour parameters of PF treated and untreated birch plywood after UV weathering of 360 h. Both the untreated controls and the plywood treated with PF resin became darker after UV weathering (decreasing ∆L*), and this effect was more pronounced for plywood treated with commercial resin D (M_w_ = 703 g/mol) at all tested concentrations (Figure 1a). This is also clearly seen in the specimen photos after UV weathering (Figure 4: UV only).

Untreated and PF resin treated plywood became redder during UV weathering (increasing Δa). The change in red colour parameter was minimal for resin B (M_w_ = 528 g/mol) while resin A and B (M_w_ = 292 and 884 g/mol) had similar changes compared to the untreated plywood. Resin D (M_w_ = 703 g/mol) treated plywood at all tested concentrations had the highest changes in redness parameter (Figure 1b). 

For all PF treated specimens, the yellowness (Δb) parameter increased similarly to between 11 and 13 units after 360 h, while untreated plywood became less yellow after UV weathering reaching 9 units after 360 h of exposure (Figure 1c).

The total colour change (Figure 1d: ΔEab) for all PF treated plywood specimens was between 12 and 18 units after 360 h of exposure. Resins A, B and C (M_w_ = 292, 528 and 884 g/mol) had lower ΔEab values (12–13 units) compared to commercial resin D (M_w_ = 703 g/mol), at all tested concentrations (15–18 units). Resin D-treated plywood did not show major differences in colour change between different concentrations, D15 showing the highest colour difference. For all PF-treated specimens, colour changes under the influence of UV light occured most rapidly in the first 24 h, after which the rate of change slowed down considerably. For untreated plywood, the change in colour parameters reached a maximum during the first 48 h and then decreased slightly further during the testing period.

### 3.2. Artificial Weathering (UV + Water Spray)

**Figure 2:**
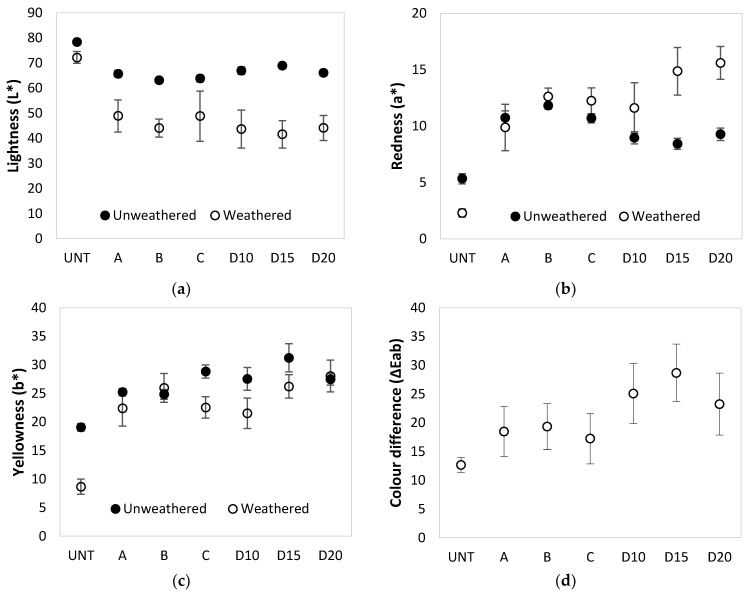
Changes in colour of untreated (UNT) and PF resin treated plywood after exposure to UV light and water spray after 1000 h in QUV camera: (**a**) Changes in the CIE parameter L* (lightness); (**b**) Changes in the CIE parameter a* (red-green); (**c**) Changes in the CIE parameter b* (yellow-blue); (**d**) Colour difference parameter ΔEab.

In order to recreate so called ”real-life” conditions, artificial weathering using a combination of UV radiation and moisture spraying was used. Moisture spraying recreated the effect of rain, mist and dew. Results presented in Figure 2 show colour parameters of PF treated and untreated birch plywood after UV weathering and water spraying, after a total test time of 1000 h.

After 100 h of UV weathering and water spraying, plywood treated with PF resin as well as untreated plywood controls became darker (L* decreased) compared to unweathered reference specimens. For PF resin-treated specimens, lightness decreased by 15–27 units, but for untreated plywood by only 6 units (Figure 2a). For untreated plywood after UV and water spraying, the redness parameter (a*) decreased by 3 units and yellowness (b*) by 10 units (Figure 2b,c). For plywood treated with resins A and B (M_w_ = 292, 528 g/mol), the redness and yellowness parameters were affected negligibly, while for resin C (M_w_ = 884 g/mol) only yellowness decreased. For plywood treated with resin D (M_w_ = 703 g/mol), redness increased by 2–6 units at all concentrations, while yellowness was only affected for 10 and 15% solid content concentration treatments (b* decreased by 5–6 units). The smallest changes in colour (ΔEab) after UV + water spray were observed for untreated plywood (13 units), for resins A, B and C, the changes reached 17–19 units, but the largest changes (23–28 units) were for resin D treated specimens at all tested concentrations (Figure 2d). A similar trend was observed for total colour change after only UV weathering.

Visual assessment of the specimens showed that the untreated plywood turned gray with surface cracking after 1000 h of UV and water spraying. All PF resin treated specimens acquired an uneven brownish colour with some shades of grey that differed considerably from the original colour of the specimens prior to weathering (Figure 4: UV + water spray). As a result of UV light and water spraying, PF resin that was not fixed within the wood cell wall leached to the plywood surface, causing uneven discolouration patterns during simulated weathering. Kielmann and Mai [15] have tested PF treated (25% (*w*/*w*) aqueous solution) beech (*Fagus sylvatica* L.) wood boards with and without coatings. After UV and water spray PF treated beech surface became darker brown, however the total colour difference (ΔEab) for PF treated wood was similar to untreated wood reaching 25–30 units.

Our data after artificial weathering with only UV light and then with UV and water spraying contradicted general information available in literature that suggests treatment with PF resin significantly improves the colour stability of the wood material [9,15,16,17,18]. Data obtained in this study shows that the total colour difference of PF treated specimens is similar (in case of only UV weathering) or even greater than that of untreated plywood.

### 3.3. Weathering under Outdoor Conditions

**Figure 3:**
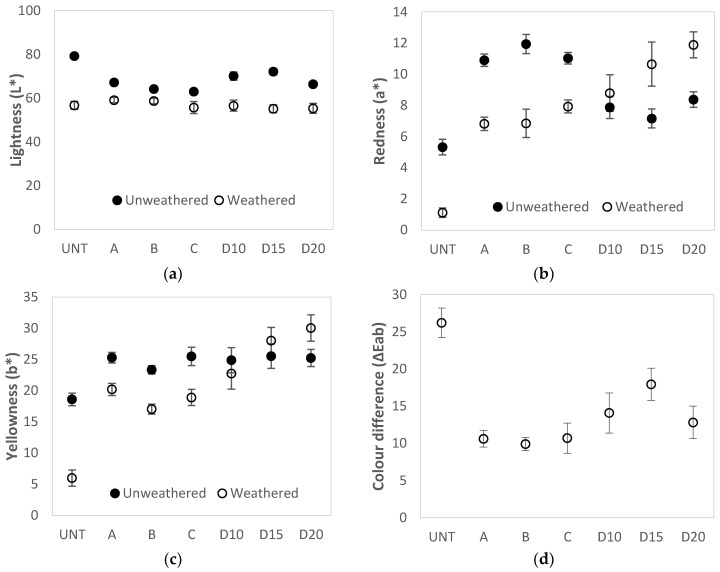
Changes in colour of untreated (UNT) and PF resin treated plywood after exposure to outdoor conditions for 3 months: (**a**) Changes in the CIE parameter L* (lightness); (**b**) Changes in the CIE parameter a* (red-green); (**c**) Changes in the CIE parameter b* (yellow-blue); (**d**) Colour difference parameter ΔEab.

Real, outdoor conditions test the ability of a material to resist weathering and discolouration processes most accurately. Therefore, untreated and PF resin treated plywood was also tested outdoors. The results presented in Figure 3 show colour parameters of PF resin treated and untreated birch plywood after 3 months of weathering in real, outdoor conditions. Here, both untreated and PF resin-treated plywood became darker (L* decreased) after weathering. For PF-treated specimens, lightness decreased by 5–17 units, while for untreated plywood, lightness decreased by 23 units (Figure 3a). For untreated plywood after outdoor exposure, the redness parameter (a*) decreased by 4 units and yellowness (b*) by 13 units, with an overall grey discolouration. For plywood specimens treated with PF resins A, B and C (M_w_ = 292, 528 and 884 g/mol), redness decreased by 3–5 units and yellowness by 5–6 units. For all specimens treated with resin D (M_w_ = 703 g/mol), the opposite tendency was observed and redness increase was 1–3 units while yellowness 2–5 units (Figure 3b,c). As a result, the largest changes in colour (ΔEab) after outdoor weathering were found for untreated plywood (26 units). For resins A, B and C, the colour changes reached approximately 10 units, but for specimens treated with resin D at all concentrations, the changes reached 12–18 units (Figure 3d). Comparable results were obtained by Evans et al. [17], who exposed PF treated radiata pine veneers in outdoor weathering for 2000 h. They concluded that higher resin loading in wood causes higher total colour changes after weathering. Only 10% of PF solution treated specimens showed lower total colour difference (ΔEab) compared to untreated wood while 20 and 30% treatments had similar values.

Under outdoor weathering conditions, PF resin-treated plywood specimens showed considerably better colour stability than untreated plywood compared to artificial conditions. This suggests that artificial weathering data do not reflect the actual properties of the material in real-use conditions.

**Figure 4:**
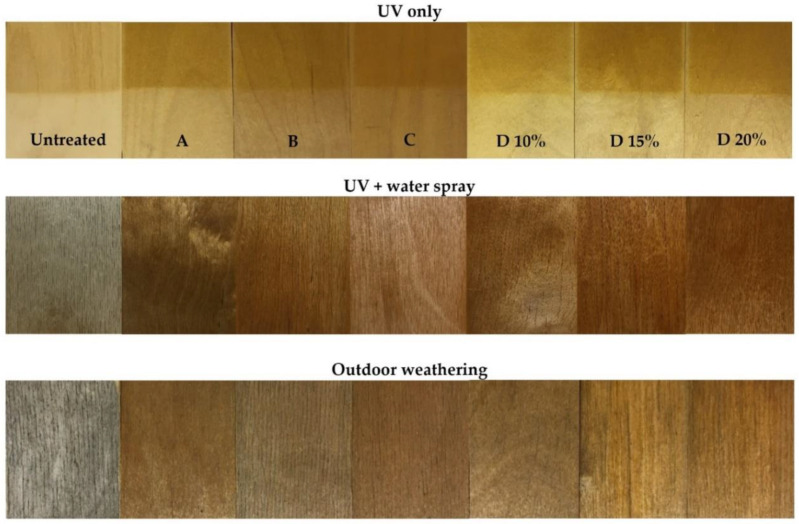
Untreated and PF resin treated plywood photo fixation after weathering with UV only, UV + water spray and in outdoor conditions for 3 months.

After three months of outdoor exposure, untreated plywood specimens turned grey with surface cracking as well as mould and blue stain fungal growth (Figure 4: Outdoor weathering). Specimens treated with PF resins A, B and C also show cracks, mould and blue stain on the surface after outdoor exposure. However, their intensity was lower and the total colour change was less pronounced. Resin D treated specimens (all solid content concentrations) changed colour the most. Mould and blue stain fungal growth developed within surface cracks.

### 3.4. Growth of Wood Discolouring Fungi on PF Treated Plywood

In outdoor aboveground conditions, wood materials are affected not only by abiotic natural weathering through UV radiation and moisture, but also though biotic damage caused by fungi and mould growth. Other anthropogenic sources, such as air pollution, can also deteriorate the surface appearance of wood. Table 4 below shows the growth rate of mould and blue stain fungi on PF resin treated plywood and untreated reference plywood throughout the 3-month outdoor exposure period. After one month, no fungal growth was observed on tested specimens. The first signs of fungal growth on PF treated plywood appeared after 2 months (rated 1–3) of outdoor exposure. After 2 months more than 50% of the untreated plywood surfaces were colonised by fungi (rated 4). After 3 months all PF resin D (M_w_ = 703 g/mol) treated specimens reached rating 4 (severe growth >50%). Higher resin loading for 15 and 20% treated specimens seemed to show no protective effect against mould and blue stain on the plywood surface. 

All PF-treated specimens reached a rating of 3–4 (medium growth 26–50% to severe growth >50%) after 3 months. However, the best results were shown by specimens impregnated with resins B and C (M_w_ = 528 and 884 g/mol), for which the colour marks rated 3–3.7.

Specimens treated with resin A, B and C (M_w_ = 292, 528 and 884 g/mol) with coated edges showed greater mould and blue stain growth after 2 and 3 months. Such a tendency was not observed with the specimens treated with resin D at all tested concentrations. In our opinion, this is due to the fact that the specimens with coated edges after wetting under the influence of rain dried up much slower, thus creating a more favourable environment for the development of mould and blue stain. However, the moisture content in specimens during outdoor test was not measured and it is difficult to confirm this assertion. Our data testify that resins A, B and C are relatively well penetrated and fixed in the birch wood cell wall and WPG after leaching decreases by 1.5–2.0% (unpublished results). Maybe this might be attributed to different unfixed PF resin part leaching during interaction with water.

PF resin-treated plywood did not considerably lower growth rate of mould and blue stain compared to untreated plywood. However, we observed that mould and blue stain on the surface of PF resin treated plywood show a weaker colouration, resulting in a lower total colour change compared to untreated plywood (Figure 4 above). Surprisingly, treatment with 15 and 20% resin D solutions did not provide better surface protection for plywood against mould and blue stain. This was most likely due to the number of surface cracks that developed during the test period. All PF resin D treated specimens were more cracked compared to PF resin A, B and C treated specimens.

### 3.5. Resistance Against Soft-Rot in Unsterile Soil-Bed Test

**Figure 5:**
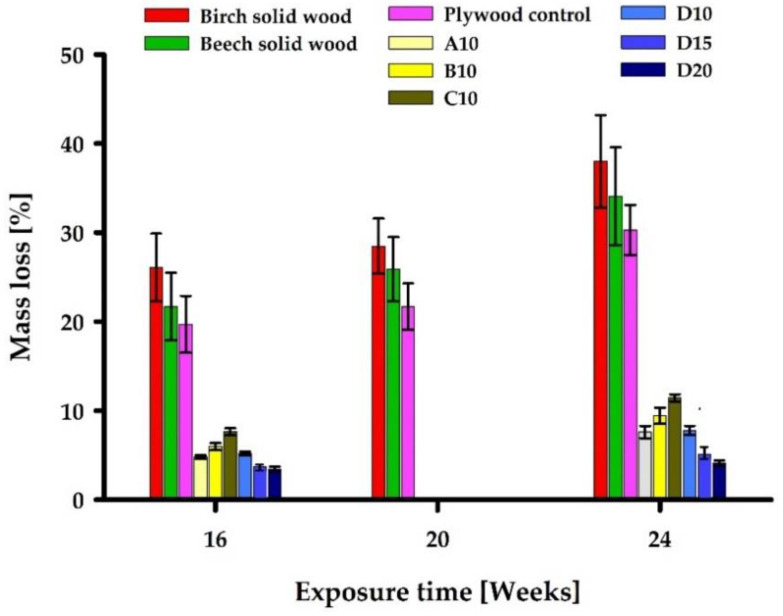
Oven-dry mass loss (ML_wood_) [%] and standard deviation [%] of PF resin impregnated plywood material as well as commercially produced untreated plywood and solid wood after 16–24 weeks of incubation in unsterile soil.

Figure 5 below shows the mean ML_wood_ and standard deviation of the untreated plywood, birch and beech solid wood and PF-treated plywood specimens. For untreated birch plywood, beech solid wood and birch solid wood specimens, measurements of ML_wood_ were performed after 16, 20 and 24 weeks of incubation. Due to limitations in the number of treated test specimens, PF resin treated specimens were only removed after 16 and 24 weeks of incubation. For all control specimens, ML_wood_ increased almost as a linear function within the test period. Among the control specimens, the highest ML_wood_ range attained was for birch solid wood (26–38%), following with beech solid wood (22–34%), while the lowest was birch plywood (20–30%).

The PF resin-treated plywood specimens showed a considerably reduced ML_wood_ compared to untreated references. The lowest ML_wood_ attained was for specimens treated with 10% solid content concentration of PF resins A and D (M_w_ = 292 and 703 g/mol), showing ML_wood_ of 4.8–7.6% and 5.3–7.8%, respectively. Specimens treated with resins B and C showed ML_wood_ of 6.0–9.4% and 7.6–11.4%, respectively. For laboratory prepared resin A, B, and C (M_w_ = 292,528 and 884 g/mol), a clear trend could be identified—use of higher molecular mass resins for veneer treatment caused higher ML_wood_ of plywood after exposure to unsterile soil. Specimens treated with commercially acquired resin D (M_w_ = 703 g/mol) at 10% solid content concentration did not fit into this trend since it showed lower ML_wood_ compared to PF resin B and C specimens treated at the same 10% solid content concentration. However, the assumption that an increase of resin loading in wood improves resistance against soft-rot was confirmed. The best results were shown by specimens treated with 15 and 20% solid content concentration solutions of PF resin D, with ML_wood_ of 3.7–5.1% and 3.4–4.1%, respectively.

#### x-Value towards Provisional Durability Rating

After 16 weeks of incubation, sufficient mean ML_wood_ (20%) of 10 reference specimens was reached for all reference specimens, exception for commercially sourced, untreated birch plywood. Subsequent incubation periods of 20 and 24 weeks also showed sufficient ML_wood_ for all untreated reference specimens. Median ML_wood_ was used to calculate an x value towards a provisional durability rating for the developed plywood material in accordance with CEN/TS 15083-2:2005 [28]. A provisional durability class (DC) rating was calculated after 16 and 24 weeks for all PF resin treated specimens against the various untreated reference control specimens (birch solid wood, beech solid wood and birch plywood). All PF treated plywood specimens could be classified as durable (DC 2) or moderately durable (DC 3). After treatment with 10% solid content concentration solutions, the best resistance to soft-rot was shown by resin A (M_w_ = 292 g/mol) treated specimens with DC 2 against birch solid wood after 16 and 24 weeks (lowest x value: Table 5). Plywood specimens treated with resins B and C (Mw = 528 and 884 g/mol) showed DC 3 against all controls at 16 and 24 weeks. Resin D (Mw = 703 g/mol) showed DC 2 relative to birch solid wood after 24 test weeks. Solid content concentration increases of resin D at 15 and 20% improved the resistance to soft-rotting fungi with specimens showing DC 2 against all reference materials after 16 and 24 weeks (Table 6).

## 4. Conclusions

In the Introduction we assumed that the weathering stability and resistance to biotic degradation agents of wood treated with low molecular weight PF resin would be improved, especially by using lower molecular weight PF resins, which in-turn increase resin loading in the wood cell wall.

Our results show that it is not possible to compare weathering processes that take place under artificial conditions with processes that take place under real, outdoor conditions. Even the combination of UV and water spraying under artificial conditions was not able to simulate similar material weathering processes as those achieved outdoors.

Birch veneers that were treated with laboratory synthesized PF resins A, B, and C (M_w_ = 292, 528 and 884 g/mol) prior to plywood production behaved better in both simulated and outdoor weathering conditions compared to plywood veneers treated with commercially sourced PF resin D. The assumption that increasing the concentration of resin in wood improves its weathering stability was not confirmed. Furthermore, it was also not possible to determine a clear trend of how mean M_w_ of a particular resin affects UV stability. Theoretically, resins with a higher molecular weight penetrate the wood cell wall less, so their concentration on the wood surface is potentially higher. Therefore, weathering stability of specimens treated with higher molecular weight resins could be expected to be better. However, our results do not support this theoretical consideration.

Resins A and D (M_w_ = 292 and 703 g/mol) seemed to be the most suitable for protection against soft-rot in the adapted unsterile soil bed test carried out in this study. For plywood treated with laboratory synthesized PF resins A, B, and C (Mw = 292, 528 and 884 g/mol), results suggest that the use of higher M_w_ resin increase ML wood after incubation in unsterile soil under various incubation periods. However, no clear correlation between M_w_ and ML_wood_ could be established when comparing all four resins used in this study-plywood veneers treated with resins A, B, C and D, at 10% solid content concentration. Specimens treated with resin D showed better resistance against soft-rot decay compared to resin B and C. It was confirmed that an increase of resin D loading in wood improves resistance against soft-rot.

## Figures and Tables

**Table 1:** Characteristic parameters of PF resins used in the study.

Resin	Viscosity (mPas)	Solid Content (%)	M_n_ (g/mol)	M_w_ (g/mol)	Dispersity Q = (M_w_/M_n_)	Free Formaldehyde (%)	pH
A	75	49.4	220	292	1.327	0.6	10.0
B	125	50.0	338	528	1.562	0.4	10.3
C	282	49.7	467	884	1.892	0.5	10.4
D	216	55.9	414	703	1.698	<0.8	9.4

**Table 2:** Veneer WPG after treatment with PF resin solutions.

Resin Treatment	A 10%	B 10%	C 10%	D 10%	D 15%	D 20%
WPG (%)	14.6 ± 1.8	13.2 ± 1.6	13.9 ± 1.5	12.5 ± 1.0	19.9 ± 3.3	27.5 ± 1.8

**Table 3:** Weather data for the outdoor, aboveground weathering test site for the 3-month test period obtained from ©weatheronline.co.uk.

Month	Mean Rainfall [mm]	Min–Max Temperature Range [°C]	Relative Humidity Range [%]	UV Index Range
June 2020	5	15–30	50–80	6–7
July 2020	6	10–28	55–90	5–6
August 2020	12	11–28	55–90	4–5
September 2020	12	7–24	55–90	2–4

**Table 4:** Mould and blue stain growth marks on PF resin treated plywood in outdoor, aboveground conditions tested according to EN 152:2011 [33].

Time (Months)		Resin Treatment						
		Untreated	A	B	C	D 10%	D 15%	D 20%
Coated edges	1	0.0	0.0	0.0	0.0	0.0	0.0	0.0
	2	4.0	3.0	2.0	2.0	2.0	2.0	2.0
	3	4.0	4.0	3.7	3.7	4.0	4.0	4.0
Uncoated edges	1	0.0	0.0	0.0	0.0	0.0	0.0	0.0
	2	4.0	2.0	1.0	2.0	2.0	2.0	2.0
	3	4.0	3.3	3.3	3.0	4.0	4.0	4.0

**Table 5:** x values calculated according to CEN/TS 15083-2 [28] for the developed plywood material against various untreated reference wood materials for 16 and 24 weeks of incubation in unsterile soil.

Wood Material	Reference Material and Exposure Time [Weeks]					
	Birch Solid	Beech Solid	Untreated Plywood	Birch Solid	Beech Solid	Untreated Plywood
	16	16	16	24	24	24
A10	0.20	0.23	0.25	0.20	0.22	0.26
B10	0.25	0.28	0.31	0.24	0.26	0.31
C10	0.31	0.36	0.40	0.29	0.32	0.38
D10	0.22	0.25	0.27	0.20	0.22	0.26
D15	0.15	0.17	0.18	0.13	0.14	0.17
D20	0.14	0.15	0.17	0.11	0.12	0.14

**Table 6:** Durability class ratings according to CEN/TS 15083-2:2005 [28] after 16 and 24 weeks of incubation in unsterile soil.

Wood Material	Reference Material and Exposure Time [Weeks]					
	Birch Solid	Beech Solid	Untreated Plywood	Birch Solid	Beech Solid	Untreated Plywood
	16	16	16	24	24	24
A10	2	3	3	2	3	3
B10	3	3	3	3	3	3
C10	3	3	3	3	3	3
D10	3	3	3	2	3	3
D15	2	2	2	2	2	2
D20	2	2	2	2	2	2
